# Case of Hodgkins Disease

**Published:** 1915-02

**Authors:** L. C. Rouglin

**Affiliations:** 829-30 Candler Building


### CASE OF HODGKINS DISEASE.

By L. C. Rouglin, M. D.

Patient: J. B. Age 10 years. Resident Atlanta, Ga.

Family History: Negative.

Past History: Had the usual diseases of childhood; at age of 18 months had "pneumonia," made a complete recovery. Had no other acute illness.

Four and a half years ago, patient began getting pale, weak, and at the same time noticed a small enlargement on left side of the neck, and complained of general pains over body. This was diagnosed as "rheumatic pains," but subsequently as the swelling continued to increase, this diagnosis was changed to "mumps." Patient was later referred to me as the "mumps" was not getting better.

PAST OBSERVATION: When first seen by me, patient was five and a half years old, of good size for his age, fairly well developed, looked rather pale, complained of being weak, eyes and face looked puffy and bloated, and had a large glandular tumor on left side of neck, extending from clavicle to angle of jaw, skin over tumor was pale and freely movable, on palpation tumor was found to consist of several nodular masses, all freely movable, of a semi-hardened consistency, and not painful on pressure.

LUNGS AND HEART: Negative.

SPLEEN: No apparent enlargement.

LIVER: Percussion area normal.

TEMPERATURE: Normal.

The Von Pirquet and Moro tests were negative.

No urinalysis or blood test was made by me at that time.

A diagnosis of Hodgkins Disease was made, and he was put on Syr. Ferri Iodidi in cod liver oil, and X-Ray exposures made to the gland. After six weeks of this treatment the gland returned to normal and stayed normal for several months. On the return of the swelling he was placed under the observation of another physician, a diagnosis of "tubercular cervical adenitis" was made, and he was given tuberculin injections. At this time patient was running a temperature of 99 to 100 F. The treatment did not have any effect, the other glands of the neck became involved, and he was referred to a surgeon for operation.

Thrice he was operated upon, the glands returning each time; after the last operation the surgeon informs me that a diagnosis of Hodgkins Disease was made. The patient was then taken to Mt. Sinai Hospital in New York, where a large gland was removed by Dr. Berg, and a histological examination of same made by Dr. Mandlebaum, and the diagnosis of Hodgkins Disease was fully confirmed.

The patient again came under my observation on December 9th, 1914. He was very much emaciated, looked extremely pale, face bloated, swellings on both sides of neck, large as a man's fist, in fact, he looked deformed a marked swelling to the left side of the mediastinal space, bulging out between the intercostal space, patient was very weak, unable to walk more than a short distance, could not sleep, had marked difficulty in swallowing or breathing. Well defined murmurs of valvular insuffiiciency could be heard at mitral and aortic openings, and a general haemic murmur could be heard over the whole praecordial area. There was marked hyperplasia of the mucous membrane of the mouth, pharynx and larynx, and glandular enlargements in groin.

SPLEEN: Enlarged.

LIVER: Area of dullness markedly increased.

TEMPERATURE: 99 F.

URINALYSIS: Negative.

HAEMATOLOGY: Examination made by Dr. C. W. Gould, of Atlanta Clinical Laboratory December 17, 1914.

White cells, 29,000.

Polymorphonuclear Leucocytes, 37.6%.

Large Lymphocytes, 44%.

Small Lymphocytes, 6%.

Transitional, 7.8%.

Eosinophyles, 4.2%.

Basophyles, .4%.

Red Cells, 5,312.000.

Second examination made by Dr. Gould January 5, 1915.

Hemoglobin, 65%.

Polymorphonuclear Leucocytes, 57.5%.

Large Lymphocytes, 11.5%.

Small Lymphocytes, 23.5%.

Transitional, 10%.

Eosinophyles, 2.5%.

Diagnosis: Hodgkins Disease: Differentiation from tubercular adenitis by absence of pain, fever, no cessation of glands, no tubercular focus elsewhere nor has any developed since the progress of this disease in this patient, negative tuberculin tests, further confirmed by the hematological and histological examinations made.

Etiology: Students of this disease have been divided in their opinion as to the etiological factor of this disease, others that it is of infectious origin. Sternberg maintains that it is due to the toxins of tubercle bacilli.

It is now generally considered that it is due to an infectious organism, a diphtheroid bacillus, isolated by Bunting and Yates in 1913. Inoculation experiments with this organism on monkeys produced glandular swellings resembling Hodgkins Disease; in some cases autogenous vaccines were used with promising results. This organism, however, has not been found constant in the glands, and Dr. Ernest Irons, in a letter to me dated the 10th inst. states, "Personally, I feel that the etiologic role of the gram positive bacillus found in nodes of Hodgkins Disease is not definitely proved." Recent inoculation experiments with this organism in Ann Arbor resulted in the production of a glandular swelling resembling Hodgkins Disease, and in others a splenic condition resembling Banti's Disease, a similar organism having been isolated from a spleen in Banti's Disease, suggesting the possibility of Banti's and Hodgkins Disease both being produced by the same organism, and that Banti's Disease is possibly a modified form of Hodgkins Disease.

Coley maintains that it is a form of Lympho-sarcoma, and has treated some cases with Coley's toxins, in one case a cure was obtained after five months' treatment, the patient remaining well when observed three years later.

Prognosis: Grave. Average duration two to five years, usually carried off by subsequent complication with tuberculosis, or from anaemic cachecsia, or die as a result of the glandular pressure upon the vital organs.

Treatment: As carried out in this case.

General: Good, nutritious diet, augmented by buttermilk between meals, careful attention to movement of bowels, sponge bath and alcohol rub daily, insistence that teeth be properly cleansed after each meal and on arising in the morning, graduated amount of exercise in the open air and sunshine.

Internal: Bone marrow and Iron three times a day.

Injections of sodii cacodylate begun with ¾ grain daily, and gradually increased, until now patient gets nine grains a week.

Local: X-Ray exposures to the glands, daily at first, and now twice a week, local massage with a 5% Iodine ointment at night.

Results: Since December 9 patient gained eight pounds in weight, has good appetite, feels strong, sleeps sound, has no difficulty in breathing or swallowing, while the glands are rapidly disappearing. This treatment will be continued until complete subsidence of the disease is procured. Should there be a later return, Coley's vaccines, or an autogenous vaccine will be tried.

829-30 Candler Building.

Read and case presented before the Fulton County Medical Society, January 21, 1915.

